# Increased levels of soluble interleukin-6 receptor and CCL3 in COPD sputum

**DOI:** 10.1186/s12931-014-0103-4

**Published:** 2014-09-04

**Authors:** Arjun K Ravi, Shruti Khurana, Jonathan Lemon, Jonathan Plumb, George Booth, Louise Healy, Matthew Catley, Jørgen Vestbo, Dave Singh

**Affiliations:** Medicines Evaluation Unit, University Hospital of South Manchester NHS Foundation Trust, University of Manchester, Manchester, UK; Manchester Academic Health Science Centre; University Hospital South Manchester NHS Foundation Trust; NIHR Respiratory and Allergy Clinical Research Facility, University of Manchester, Manchester, UK; UCB, Slough, Berkshire UK; Department of Cardiology and Respiratory Medicine, Hvidovre Hospital and University of Copenhagen, Copenhagen, Denmark

**Keywords:** COPD, Sputum, Two-step sputum processing, Interleukin-6, sIL-6R, CCL3

## Abstract

**Background:**

COPD patients have increased numbers of macrophages and neutrophils in the lungs. Interleukin-6 (IL-6) trans-signaling via its soluble receptor *sIL-6R*, governs the influx of innate immune cells to inflammatory foci through regulation of the chemokine *CCL3*. We hypothesized that there would be enhanced levels of IL-6, sIL-6R and CCL3 in COPD sputum.

**Methods:**

59 COPD patients, 15 HNS and 15 S underwent sputum induction and processing with phosphate buffered saline to obtain supernatants for IL-6, sIL-6R and CCL3 analysis. Cytoslides were produced for differential cell counting and immunocytochemistry (COPD; n = 3) to determine cell type surface expression of the CCL3 receptors CCR5 and CCR1.

**Results:**

COPD patients expressed higher levels (p < 0.05) of sIL-6R and CCL3 compared to controls (sIL-6R medians pg/ml: COPD 166.4 vs S 101.1 vs HNS 96.4; CCL3 medians pg/ml: COPD 117.9 vs S 0 vs HNS 2.7). COPD sIL-6R levels were significantly correlated with sputum neutrophil (r = 0.5, p < 0.0001) and macrophage (r = 0.3, p = 0.01) counts. Immunocytochemical analysis revealed that CCR5 and CCR1 were exclusively expressed on airway macrophages.

**Conclusion:**

Enhanced airway generation of sIL-6R may promote IL-6 trans-signaling in COPD. Associated upregulation of CCL3 may facilitate the recruitment of macrophages into the airways by ligation of CCR1 and CCR5.

**Electronic supplementary material:**

The online version of this article (doi:10.1186/s12931-014-0103-4) contains supplementary material, which is available to authorized users.

## Introduction

Chronic Obstructive Pulmonary Disease (COPD) arises due to an abnormal inflammatory response to inhaled noxious particles, such as cigarette smoke [1]. There are increased numbers of neutrophils and macrophages in the airways of COPD patients [2]. These cells organize the innate immune response, and contribute to inflammation through the secretion of cytokines, chemokines, elastolytic enzymes and reactive oxygen species [3,4].

The cytokine interleukin-6 (IL-6) exerts pro-inflammatory effects through the activation of JAK-STAT (Janus Kinase – Signal Transducer and Activator of Transcription) signaling [5]. IL-6 initiates the acute phase response, stimulating the secretion of C-reactive protein (CRP) [6]; IL-6 and CRP levels are increased during acute exacerbations of COPD [7]. There is also evidence that increased serum and broncho-alveolar lavage (BAL) IL-6 levels are associated with lower lung function in COPD [8], suggesting a role for IL-6 in the chronic inflammatory processes that cause disease progression.

The IL-6 receptor has two components: an IL-6 binding domain (IL-6R) and a membrane bound signal transducing element (gp130) [9]. IL-6R can be proteolytically cleaved from the cell membrane by the metalloproteinase ADAM17 (A Disintegrin and Metalloproteinase-17) to generate a soluble form (sIL-6R) [10]. IL-6 bound to sIL-6R forms a complex that binds to cell surface gp130 to initiate signal transduction, a process known as trans-signaling [11]. The role of IL-6 in inflammation is critically dependent on sIL-6R levels; IL-6 trans-signaling controls macrophage recruitment during acute inflammation [12]. Furthermore, IL-6 signaling can be upregulated by increased sIL-6R levels without a change in IL-6 levels [12].

CCL3 is monocyte and macrophage chemoattractant [13], acting through the receptors CCR1 and CCR5. There is evidence that CCL3 levels are increased in the lungs of COPD patients; CCL3 gene expression is increased in COPD bronchial epithelial cells compared with healthy controls [14,15] and a small study recently showed increased sputum supernatant CCL3 levels in COPD patients [15]. CCL3 regulates the expression of IL-6; CCL3 stimulation increases IL-6 expression in peritoneal macrophages [16]. Additionally, IL-6 knock-out mice have significantly reduced CCL3 and macrophage counts in BAL after the intra-tracheal administration of bleomycin [17]. The expression and activity of IL-6 and CCL3 appear to be closely associated, and involved in the regulation of macrophage numbers during inflammation [17].

We hypothesized that IL-6, sIL-6R and CCL3 levels are increased in the airways of COPD patients. We therefore investigated the levels of these proteins in induced sputum from COPD patients compared to controls. We also studied the expression of the CCL3 receptors, CCR1 and CCR5, on airway macrophages and neutrophils to determine the target immune cell for this chemokine in the lungs of COPD patients.

## Methods

### Subjects

59 COPD patients, 15 smokers (S) and 15 healthy non-smokers (HNS) were recruited from primary care between January and August 2011. COPD patients had been diagnosed according to current guidelines [1]. S with >10 pack year history and HNS had normal lung function. Patients with a history of asthma, respiratory tract infection or COPD exacerbation within the preceding six weeks were excluded. The research was approved by a local ethics committee (Greater Manchester (East): Approval number 05/Q140241) and all participants provided written informed consent.

### Study design

COPD patients performed the following procedures on a single study visit: spirometry, body plethysmography, transfer factor/transfer coefficient, 6 minute walk test (6MWT), St George’s Respiratory Questionnaire (SGRQ) [18], modified Medical Research Council dyspnoea scale (mMRC) [19], COPD Assessment Tool (CAT) [20], Medical Research Council chronic bronchitis questionnaire [21] and sputum induction. S and HNS underwent spirometry and sputum induction only. 6 HNS underwent venesection to provide blood for monocyte isolation (demographic data presented in Additional file 1: Table S1). Permission was obtained for the utilization of SGRQ and CAT (Additional file 2: Terms of use).

### Pulmonary function

Maximum expiratory flow volume measurements were performed in triplicate on a Vitalograph gold standard wedge bellows spirometer; we recorded the highest FEV1 (forced expiratory volume in 1 second) and FVC (forced vital capacity). Readings were repeated after 20 minutes following the inhalation of 200 μg salbutamol via spacer. FRC (functional residual capacity), TLC (total lung capacity) and IC (inspiratory capacity) were measured pre-bronchodilator using a constant volume plethysmograph (Sensormedics Vmax Encore V62J body plethysmograph) according to ERS standards [22]. Carbon Monoxide Diffusion Capacity (DLCO) and Transfer coefficient (KCO) were determined using the Sensormedics Vmax Encore V62J body plethysmograph) according to ERS standards [23]. The 6MWT was performed according to ATS standards [24].

### Sputum induction and processing

Sputum was induced and processed by the two-step method (initial phosphate buffered saline (PBS) wash followed by the addition of Dithiothreitol (DTT)) as previously described [25]; this method avoids the unwanted effects of DTT on immunoassays applied to the supernatants. Cytoslides were produced to determine differential cell count and for immunocytochemistry.

For differential cell counts, cytoslides were air dried, fixed with methanol and stained with Rapi-diff (Triangle, Skelmersdale, UK). Four hundred non-squamous cells were counted and the results expressed as a percentage of the total cell count (TCC), and a total cell count per gram of sputum (TCC/g). Unfixed cytoslides were stored frozen at −80°C prior to immunocytochemistry (ICC).

### Cytokine measurements

Levels of IL-6, sIL-6R and CCL3 in PBS processed sputum supernatant were measured using Meso Scale Discovery® (MSD) immunoassay (Gaithersburg (MD)-USA). Analysis was performed at UCB (Slough, UK). The lower limits of quantification (LLOQ) were: IL-6 (1.6 pg/ml), sIL-6R (0.1 pg/ml) and CCL3 (15.0 pg/ml). Levels below the LLOQ were assigned values of 0 pg/ml. It has previously been shown that the measurement of IL-6, sIL-6R and CCL3 in PBS processed sputum using MSD provides satisfactory recovery of spiked analyte [25].

### Immunocytochemistry

ICC was performed on sputum cytoslides from 3 COPD patients to identify cell-type specific expression of the binding receptors for CCL3 (CCR1 and CCR5). Cells were fixed in 4% paraformaldehyde (Sigma, Poole, UK). Rabbit anti-human CCR1 and CCR5 (AbCam, Cambridge, UK) primary antibodies diluted in 1.5% normal serum (Vector Labs, Peterborough, UK) were applied overnight at 4°C. Endogenous peroxidase was quenched by incubating sections in 3% H_2_O_2_ (hydrogen peroxide, Sigma) for 30 minutes at room temperature. CCR1 and CCR5 were detected using biotinylated goat anti-rabbit immunoglobulin (IgG) secondary antibody (Vector Labs) in conjunction with an avidin-biotin peroxidase complex (Vector Labs). CCR1 and CCR5 were visualised using 3,3′-diaminobenzidine (DAB) substrate and counterstained with modified Gill’s haematoxylin. Omission of primary antibody from staining protocol and substitution of primary antibody with an isotype control antibody (Vector Labs) were used as negative controls. Digital micrographs were obtained using a Nikon Eclipse 80i microscope (Nikon UK Ltd, Surrey UK) equipped with a QImaging digital camera (Media Cybernetics, Marlow, UK) and ImagePro Plus 6.0 software (Media Cybernetics). The number of cells that stained positively for CCR1 and CCR5 were determined using the manual-tag function in ImagePro Plus. 100 cells were counted per slide.

### CD14+ monocyte isolation

50 mls of blood was obtained by venipuncture and collected in a heparinized tube. Peripheral blood mononuclear cells (PBMC) were isolated by Ficoll differential centrifugation [26]. CD14+ monocytes were positively selected from the PBMCs by magnetic bead isolation as previously described [26], and then suspended in either RPMI (Sigma) or PBS (for ICC and chemotaxis experiments respectively).

### CD14+ monocyte CCR1 and CCR5 surface expression

In order to establish whether CCL3 interferes with the ICC detection of CCR1 and CCR5, we performed an experiment assessing the CCR1 and CCR5 expression on CD14+ monocytes cultured in the presence of CCL3. 3 HNS donated blood for monocyte isolation.

CD14+ cells were seeded (1×10^5^ cells/well) in a 96-well plate (Greiner Bio-One, Stonehouse , UK) and incubated in the presence or absence of rh-CCL3 (recombinant human CCL3; 1 ng/ml: R&D systems, Abingdon, UK) for 45 minutes at 37°C and 5% CO_2_. Cytoslides were prepared from the cell suspensions and analysed for CCR1 and CCR5 expression by ICC as described above. Morphological analysis confirmed CD14+ cells as monocytes.

### Sputum supernatant induced monocyte chemotaxis

An experiment was performed to assess whether antagonism of CCL3 resulted in reduced migration of CD14+ monocytes towards PBS processed COPD sputum supernatant.

3 HNS donated blood for monocyte isolation. The chemotaxis assay was performed using 96-well Multiscreen™ MIC plates incorporating 5 μm pore diameter polycarbonate filter membranes (Merck-Millipore, Watford-UK) based on the method of transwell diffusion [27,28]. CD14+ monocytes (1 × 10^5^ cells/well) were applied to the upper plate and incubated with either anti-human CCR5 monoclonal antibody (mAb) (20 μg/ml, R&D Systems) or PBS alone. PBS sputum supernatant obtained from 3 individuals with COPD was pooled and diluted in a 1:10 ratio with PBS. Diluted sputum supernatant was applied to the wells of the lower plate in the presence/absence of anti-human CCL3 mAb (0.5 μg/ml, R&D Systems). The two plates were incubated separately for 60 minutes at 37°C then sandwiched together and incubated for a further 18 hours. Migrated cells in the lower plate were collected, incubated with lysis buffer containing Quant-IT PicoGreen fluorescent dsDNA binding probe (Life Technologies, Paisley-UK) and analysed using a BMG LabTech FluoStar Optima optical density plate reader. CD14+ monocyte chemotaxis was expressed as a proportion (%) of maximum CD14+ monocyte migration (CD14+ cells added directly to the wells of the lower plate) [28]. All conditions were assayed in triplicate. Concentrations of the neutralizing monoclonal antibodies used were determined based on data obtained from neutralization experiments (ND_50_) conducted by the manufacturer.

### Statistical analysis

Sputum supernatant protein data were non-parametric, while sputum cell count data was parametric with the exception of eosinophil counts. When 3 groups were compared, one-way analysis of variance (ANOVA) or Kruskal-Wallis tests were performed followed by the respective application of Tukey’s or Dunn’s post-tests. Mann–Whitney U tests were applied where appropriate. The effects of CCL3 and CCR5 inhibition on % CD14+ monocyte chemotaxis were analysed using paired parametric t-tests. Each individual value from triplicate recordings using monocytes from 3 blood samples was included in the analysis. Univariate correlations of the data were performed using the Spearman rank test. P values <0.05 were considered to be significant. Analysis was performed using Prism version 5.0 (GraphPad software, San-Diego, USA) and SPSS version 19.0 (SPSS Inc., Chicago, USA).

## Results

### Subject demographics

The clinical characteristics of the study subjects are summarized in Table 1. The COPD patients were significantly older than the control groups, although the smoking pack year history was similar in COPD patients and S. There were 12 patients with GOLD stage I disease severity, 35 GOLD stage II, 11 GOLD stage III and 1 GOLD stage IV.

**Table 1:** **Demographic details of study participants**

	**COPD (n = 59)**	**S (n = 15)**	**HNS (n = 15)**	**p-value**
Sex (F:M)	23:36	8:7	4:11	-
Age*	67 (45–75)	52 (38–62)	32 (19–75)	p < 0.0001
Pack years smoked*^§^	34.9 (12.5-122.2)	29 (15–67)	0	ns
Current smokers (%)	41	100	0	-
ICS users (%)	67.8	-	-	-
Chronic Bronchitis (n) (%)	35 (59%)	-	-	-
FEV1% predicted^1a^	61.6 (18.1)	95.7 (13.0)	116.7 (13.8)	p < 0.0001
FEV1/FVC %^1^*	51.0 (28.9-67.0)	74.8 (70.5-80.8)	85.7 (72.6-101.6)	p < 0.0001
FEV1 % predicted^2a^	64.5 (17.2)	98.64 (12.1)	117.9 (14.3)	p < 0.0001
FEV1/FVC %^2^*	57.1 (25.8-69.6)	73.0 (70.2-82.5)	86.1 (80.7-93.7)	p < 0.0001
IC (L)^1a^	2.2 (0.7)	-	-	-
TLC% predicted^1^*	102.3 (8.4-164.7)	-	-	-
KCO% predicted^1a^	88.1 (25)	-	-	-
6MWT distance (m)*	387 (28–540)	-	-	-
SGRQ (total)^a^	37 (19.8)	-	-	-
mMRC^a^	1.2 (1)	-	-	-
CAT^a^	17.5 (7.9)	-	-	-

*Data presented as median (range). ^a^Data presented as mean (SD). ^1^Pre-bronchodilator. ^2^Post-bronchodilator. Explanation of abbreviations: IC = Inspiratory capacity, TLC = total lung capacity, KCO = transfer coefficient, 6MWT = 6 minute walk test, SGRQ = St George’s respiratory questionnaire, mMRC = modified Medical Research Council Dyspnoea score, CAT = COPD assessment tool, ns = not significant. The statistical significance of differences between groups was determined using either ANOVA + Tukey’s multiple comparison test or Kruskal-Wallis + Dunn’s post-test (for analysis of parametric and non-parametric data respectively). ^§^Mann–Whitney U test was performed to assess the statistical significance of differences in pack-year smoking history between COPD & S.

### Sputum counts

COPD sputum samples had increased neutrophil percentage and cell count/g compared to S (p < 0.001) and HNS (p < 0.001) – see Table 2. COPD sputum samples also had increased eosinophil percentage and cell count/g compared to S (p < 0.0001) and HNS (p < 0.0001), and a reduced macrophage percentage compared to S (p < 0.05) and HNS (p < 0.05).

**Table 2:** **Sputum cell counts**

	**COPD**	**S**	**HNS**	**p-value (ANOVA)**
Neutrophil%	69.5 (23.8)*	54 (25.1)	38.7 (27.2)	0.0003
Macrophage%	26.6 (23.7)**§	48.7 (28.4)	46.3 (33.1)	0.0033
Eosinophil %^	1.3 (0–23.3)*†	0 (0–1.5)	0 (0–2.5)	<0.0001
Lymphocyte %	0.1 (0.4)	0.1 (0.5)	0.02 (0.06)	ns
TCC*10^6^	3.3 (3.6)**	2.9 (4.4)	0.7 (0.7)	ns
TCC/g*10^6^	3.5 (3)	2.9 (3.2)	2.4 (2.2)	ns
Neutrophil TCC/g*10^6^	2.6 (2.8)*†	0.3 (0.2)	0.3 (0.2)	0.0004
Macrophage TCC/g*10^6^	0.7 (0.7)	0.5 (0.8)	0.4 (0.5)	ns
Eosinophil TCC/g*10^6^^	0.03 (0–1.5) ‡¶	0 (0–0.02)	0 (0–0.01)	<0.0001
Lymphocyte TCC/g*10^6^	0.003 (0.007)	0.001 (0.002)	0.0001 (0.001)	ns

Data is presented as mean (SD) with the exception of ^^^eosinophil % and eosinophil TCC/g which are presented as median (range). Explanation of *abbreviations: TCC* Total cell count, *TCC/g* Total cell count/gram *p < 0.001 COPD vs HNS, **p < 0.05 COPD vs HNS, §p < 0.05 COPD vs S, †p < 0.001 COPD vs S, ‡p < 0.0001 COPD vs HNS, ¶ p < 0.0001 COPD vs S.

### Sputum supernatant measurements

**Figure 1:**
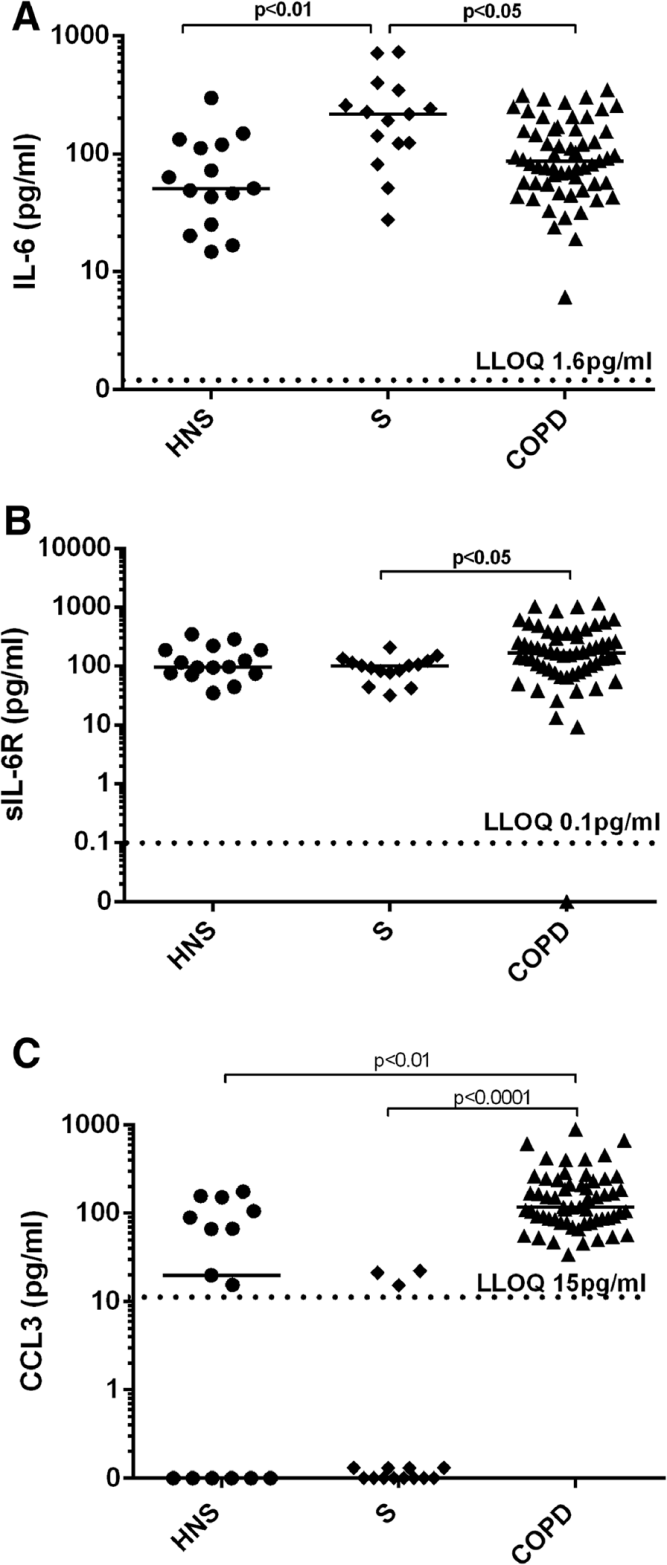
**Sputum Cytokine Concentrations.** Concentrations of **(A)** IL-6, **(B)** sIL-6R and **(C)** CCL3 in induced sputum from HNS, S and COPD patients. Each dot represents the data from an individual patient. The horizontal bar represents the median value. The dotted line represents the lower limit of quantification (LLOQ) for the respective analytes. **(B)** 1 COPD subject had sIL-6R levels registering below the LLOQ. **(C)** 6 HNS and 12 S had CCL3 levels registering below the lower limit of quantification.

Sputum supernatant levels of IL-6 were increased in S (median 217.8 pg/ml) compared to COPD patients (median 87.3 pg/ml, p < 0.05) and HNS (median 50.9 pg/ml, p < 0.01) (see Figure 1). COPD patients had numerically higher levels of IL-6 compared to HNS, but this difference did not reach statistical significance (p = 0.068). The levels of sIL-6R were significantly greater in COPD patients (median 166.4 pg/ml) compared to than S (median 101.1 pg/ml, p < 0.05), although the difference compared to HNS did not reach statistical significance (median 96.4 pg/ml, p = 0.10). COPD patients had significantly higher levels of CCL3 (median 117.9 pg/ml) compared to S (median 0 pg/ml, p < 0.0001) and HNS (median 19.9 pg/ml, p < 0.001).

**Figure 2:**
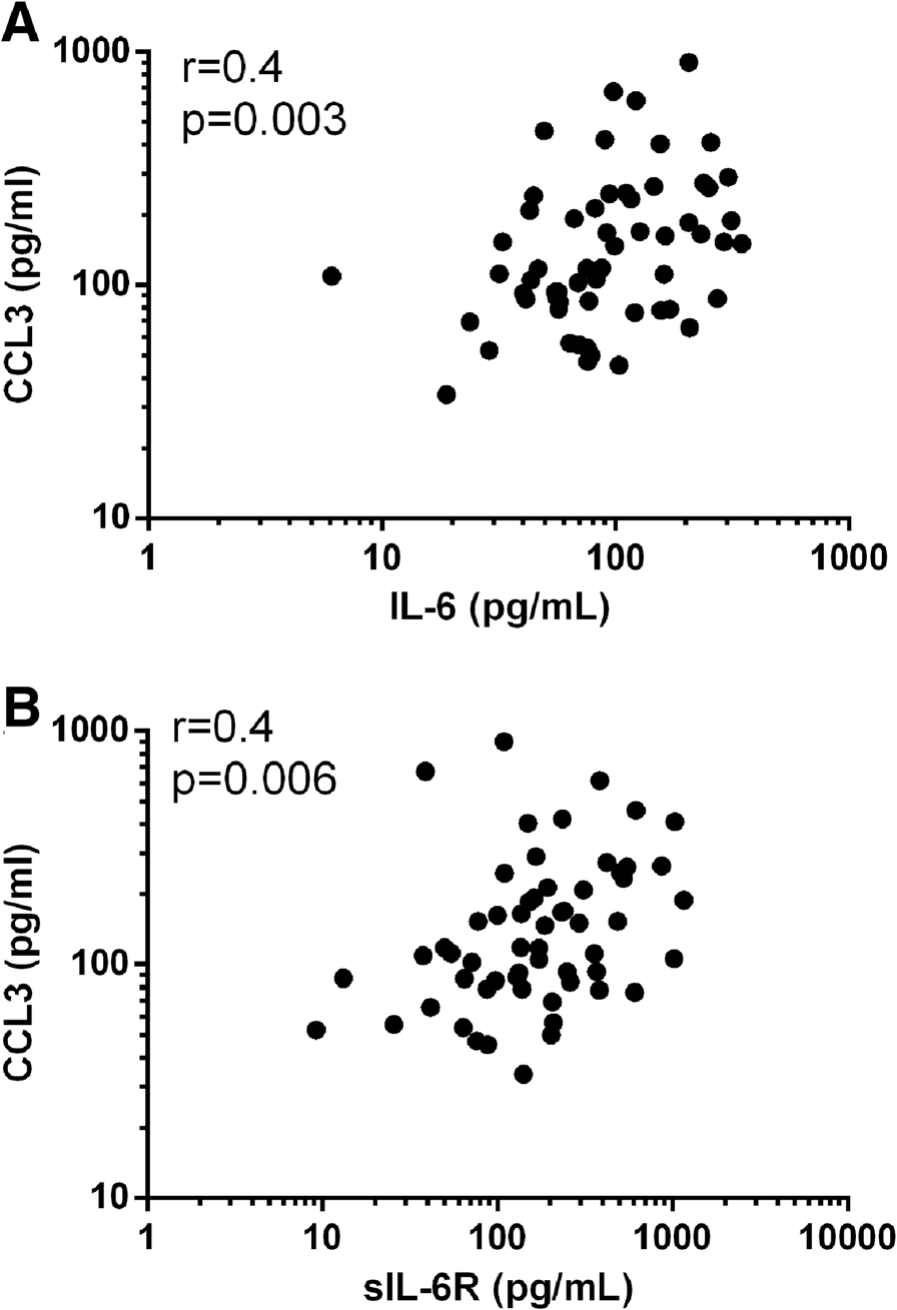
**Univariate Correlations of COPD sputum CCL3 with IL-6 and sIL-6R.** Univariate correlation between sputum **(A)** IL-6 and **(B)** sIL-6R with CCL3 in COPD patients. r represents the Spearman Rank correlation coefficient. Each dot represents the data for an individual patient.

**Figure 3:**
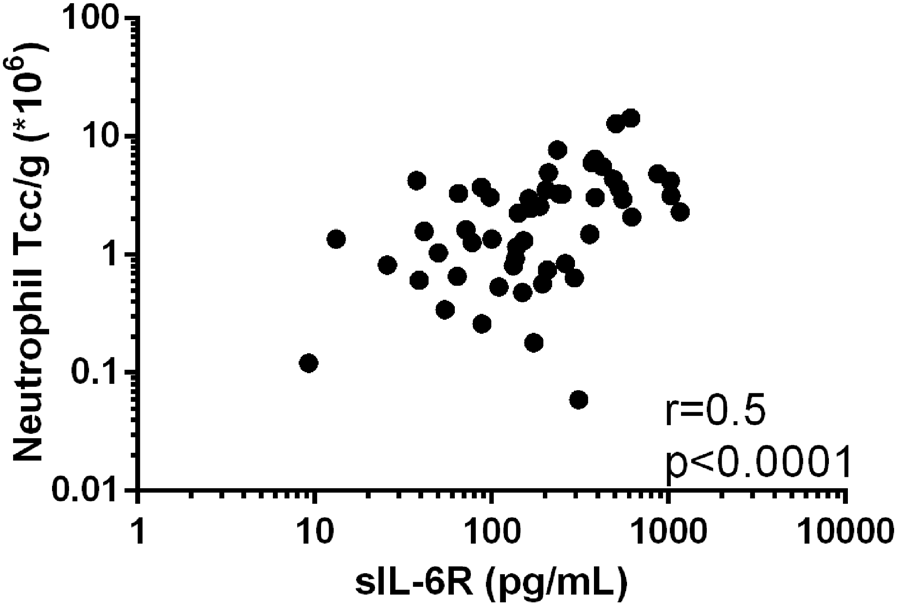
**Univariate Correlation of COPD sputum sIL-6R with sputum neutrophil TCC/g.** Univariate correlation between COPD sputum sIL-6R and neutrophil TCC/g. r represents the Spearman Rank correlation coefficient. Each dot represents the data for an individual patient.

In COPD sputum, the association between IL-6 and IL-6R levels did not reach statistical significance (r = 0.2, p = 0.06). Both IL-6 and sIL-6R had statistically significant but weak (r = 0.4), positive correlations with CCL3 levels in COPD (see Figure 2). The relationships between these supernatant proteins and sputum cells in COPD patients was also analysed; the strongest associations were observed between sputum sIL-6R levels and total cell count, and the total number of neutrophils (see Figure 3), macrophages and eosinophils (see Additional file 3: Table S2).

35 (59%) of the COPD patients in this study had chronic bronchitis. We observed that subjects with chronic bronchitis expressed numerically greater levels of all measured sputum cytokines compared to patients without chronic bronchitis; these differences were not statistically significant (see Additional file 4: Table S3). There were no significant associations between sputum IL-6, sIL-6R and CCL3 levels with FEV1% predicted, exacerbation rate, 6MWT distance, SGRQ, mMRC and CAT in COPD patients (p > 0.05 for all analysis, data not shown). The levels of these supernatant proteins also did not differ in COPD patients who were ICS (inhaled corticosteroid) users compared to those not using ICS, or in current smokers compared to ex-smokers (p > 0.05 for all comparisons, data not shown).

### Expression of CCR1 and CCR5 on sputum leucocytes

**Figure 4:**
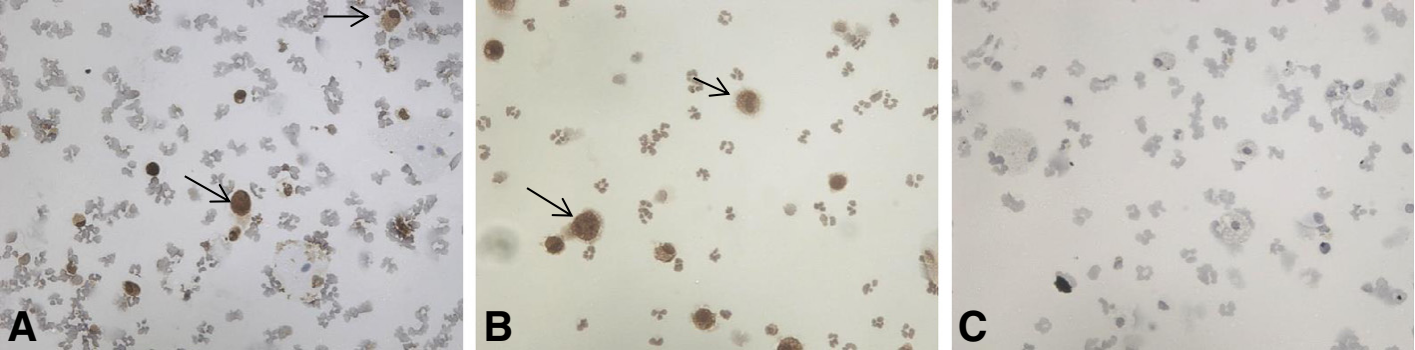
**CCR1 and CCR5 expression of sputum inflammatory cells.** Representative images of **(A)** CCR1 and **(B)** CCR5 expression on sputum inflammatory cells. Immunocytochemical labeling on sputum inflammatory cells from COPD patients (n = 3). CCR1 and CCR5 were detected using 3,3′-diaminobenzidine (DAB) substrate (brown). Arrows indicate CCR1 **(A)** and CCR5 **(B)** immunoreactive cells. **(C)** Negative control slide.

As COPD patients had increased sputum CCL3 levels, we were interested to investigate the expression of the CCL3 receptors. ICC using COPD sputum (n = 3) showed that CCR1 and CCR5 were expressed in sputum macrophages; Figure 4 shows a representative sample with positive staining for macrophages but no staining on neutrophils. All sputum macrophages expressed CCR1 and CCR5.

To confirm that the CCR1 and CCR5 antibodies bind to their respective chemokine receptors irrespective of receptor occupancy status, we used CD14+ peripheral blood monocytes isolated from HNS (n = 3); these cells expressed CCR1 and CCR5 (see Additional file 5: Figure S1). CCR1 and CCR5 expression did not change after culture with CCL3 or media alone.

### CD14+ monocyte chemotaxis

**Figure 5:**
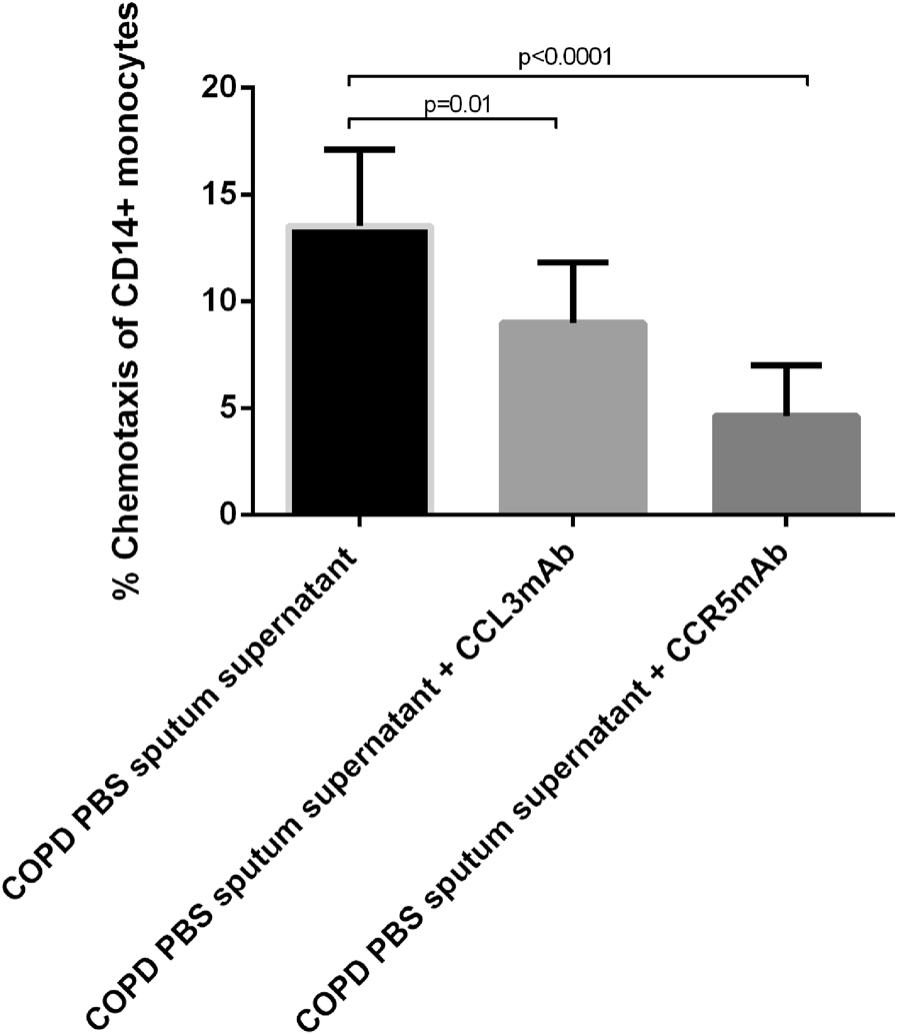
**Effect of CCL3 antagonism on migration of CD14+ monocytes towards COPD sputum supernatant.** Assessment of CD14+ monocyte migration towards COPD PBS sputum supernatant in the presence of anti-human CCL3 mAb and anti-human CCR5 mAb. Peripheral blood CD14+ monocytes were isolated from three HNS. Chemotaxis is presented as the percentage of maximum chemotaxis (CD14+ monocytes added directly to wells of the lower insert of the chemotaxis plate) and is described as mean (SD). Data represents the results from three independently conducted experiments. Statistical significance of differences in %CD14+ monocyte chemotaxis observed between control (PBS sputum supernatant alone) and experimental conditions (+CCL3 mAb or + CCR5 mAb) was determined by application of paired parametric t-tests.

The mean level of CD14+ monocyte migration towards COPD PBS sputum supernatant was 13.5% (see Figure 5). Levels of CD14+ monocyte migration were significantly reduced by anti-human CCL3 mAb (9.0%, p = 0.01) and anti-human CCR5 mAb (4.7%, p < 0.0001).

## Discussion

The levels of CCL3 in sputum supernatants were significantly increased in COPD patients compared to both S and HNS. Soluble IL-6R levels were significantly increased in COPD patients compared to S. These results indicate a potential role for IL-6 trans-signaling and CCL3 in COPD pulmonary inflammation. The CCL3 receptors CCR1 and CCR5 were expressed on airway macrophages but not neutrophils. Antagonism of CCL3 resulted in a significant reduction in CD14+ monocyte migration towards COPD sputum supernatant implicating this chemokine in the processes that control monocyte/macrophage trafficking and positioning within the lungs of COPD patients.

Previous studies of IL-6 levels within the lungs of COPD patients have omitted to study the levels of sIL-6R [7,25]. This soluble receptor is an important component of the IL-6 signaling pathway; increased sIL-6R levels alone, without a change in IL-6 levels, can upregulate IL-6 signaling [12]. We found increased sIL-6R levels in COPD patients compared to S, with the difference between COPD patients and HNS failing to reach statistical significance (p = 0.1). Nevertheless, our results suggest that IL-6 trans-signaling may be increased in the airways of COPD patients, facilitating the actions of IL-6 through JAK-STAT activation [5].

Neutrophils shed sIL-6R from the cell surface membrane following proteolytic cleavage by ADAM17 [10]. It has been demonstrated previously [29], and confirmed here, that the sputum neutrophil total cell counts are raised in COPD patients compared to controls; increased neutrophil numbers may contribute to the greater sIL-6R levels observed in COPD patients. This possibility is supported by the significant associations between sIL-6R levels and neutrophil total cell count (Figure 3).

COPD patients had significantly increased sputum absolute neutrophil counts compared to healthy controls. There was a numerical increase in the absolute sputum macrophage count in COPD patients, but this was not statistically significant. It is well known that macrophage numbers are elevated in the lungs of COPD patients compared with healthy controls [2,30]. However, in sputum samples there is a greater relative increase in neutrophil compared to macrophage absolute counts, leading to an increased percentage of neutrophils.

IL-6 levels were significantly increased in S compared to HNS, indicating that IL-6 is part of the innate immune response activation that occurs in smokers with normal lung function. Similarly, mice exposed to cigarette smoke have increased pulmonary IL-6 levels compared to air-exposed control mice [31]. IL-6 levels were lower in COPD patients compared to S. Blunting of the innate immune response in COPD patients has been demonstrated in alveolar macrophages [32,33] suggesting that persistent and long term airway inflammation leads to down-regulation of certain components of the innate immune response. There was a great spread of results for IL-6 in COPD; it is possible that IL-6 plays an important role in airway inflammation in a subgroup of COPD patients only. It is increasingly recognized that COPD comprises different phenotypes of disease [34], and IL-6 may play an important role in specific phenotypes [7,35]. It has been shown that the levels of IL-6 in BAL are highest in patients with severe COPD [8]. The majority of our patients had moderate COPD, and it is possible that increased IL-6 levels would be more apparent in patients with more severe disease. Furthermore, IL-6 in conjunction with IL-17 upregulates expression of the mucins MUC5AC and MUC5B by epithelial cells, and so IL-6 may be more important in COPD patients with mucus hypersecretion [36].

Chronic bronchitis is associated with increased airway inflammation and poorer clinical outcomes in COPD [37,38]. We observed no significant difference between COPD patients with chronic bronchitis compared to those without for IL-6, sIL-6R and CCL3 levels. Monsό [39] observed that 22% of clinically stable COPD patients with chronic bronchitis were found to have bacterial colonization of the lower airways. Increases in the airway bacterial load in COPD patients are associated with elevation in the concentrations of certain inflammatory cytokines in the sputum [40], including IL-6. We did not assess sputum microbiology, and so were unable to study the relationships between bacterial load and the levels of IL-6, sIL-6R and CCL3.

CCL3 levels were significantly elevated in sputum supernatants of COPD patients compared with healthy controls. It has previously been reported in a small study (with 29 COPD patients, 12 S and 6 HNS) which used DTT processed sputum [15] that COPD patients had higher levels of sputum CCL3 than HNS. We definitively show increased CCL3 levels in COPD sputum using a larger sample size and PBS processed sputum in order to avoid any effect of DTT on immunoassays. Increased CCL3 in COPD sputum may derive from the bronchial epithelium, as it has been reported that COPD bronchial epithelial cells express increased CCL3 mRNA levels compared with controls [14].

CCL3 is a known chemoattractant for monocytes and macrophages. Wang et al. [15] reported overall increased total CCR5 expression in COPD sputum by optical density analysis of immunohistochemistry performed on sputum cells from COPD patients and controls, but did not report the cell type expressing CCR5. We found that the CCL3 receptors CCR1 and CCR5 were exclusively expressed on sputum macrophages and not neutrophils, further implicating CCL3 in controlling macrophage chemotaxis in the airways of COPD patients. Furthermore, we demonstrated that blockade of CCL3 and its receptor CCR5 attenuated the migration of CD14+ monocytes towards COPD sputum supernatant. This confirms the role of the CCL3 present in COPD supernatant as a monocyte chemoattractant.

It has previously been reported that approximately 45% of COPD bronchoalveolar lavage neutrophils express CCR5 [41]. Pre-apoptotic neutrophils have been demonstrated to upregulate expression of CCR5 with a view to ‘soaking-up’ CCL3 as part of the resolution phase of an acute inflammatory response [41]. Therefore, even if CCR5 were expressed on the surface of neutrophils, CCL3 may not be functioning as a chemoattractant for these cells. Our finding that CCL3 levels did not correlate with sputum neutrophil counts further substantiates the notion that it is not acting as a chemoattractant for sputum neutrophils.

## Conclusion

We report increased CCL3 levels in COPD patients, and demonstrate that CCR1 and CCR5, the receptors for this chemokine, are expressed on lung macrophages. Blockade of CCL3 resulted in reduced chemotaxis of CD14+ cells towards COPD sputum supernatant. These findings suggest a prominent role for CCL3 in the upregulation of macrophage numbers seen in the lungs of COPD patients. Furthermore, sIL-6R levels were increased in COPD patients, suggesting an increase in IL-6 trans-signaling. IL-6 promotes chronic inflammation through a variety of mechanisms [42–44], and it appears that increased sIL-6R plays a role in mediating these effects in the lungs of COPD patients.

## Additional files

Additional file 1: Table S1. Depicts the demographic details of the 6HNS who donated blood for CD14+ monocyte isolation. Additional file 2:
**Terms of use.**
Additional file 3: Table S2. Depicts univariate correlatons between sputum inflammatory cells and sputum cytokines measured in COPD sputum supernatant. Additional file 4: Table S3. Depicts comparisons of sputum cytokine levels in COPD patients with chronic bronchitis compared to those without chronic bronchitis. Additional file 5: Figure S1. Depicts immunocytochemistry for CCR1 and CCR5 expression on isolated CD14+ peripheral blood monocytes in the presence/absence of CCL3.

## Abbreviations

ADAM17: A disintegrin and metalloproteinase 17
BAL: Broncho-alveolar lavage
CAT: COPD assessment tool
COPD: Chronic obstructive pulmonary disease
DAB: 3,3′-diaminobenzidine
DLCO: Carbon monoxide diffusion capacity
DTT: Dithiothreitol
FEV1: Forced expiratory volume in 1 second
FVC: Forced vital capacity
GSK: GlaxoSmithKline
H_2_O_2_: Hydrogen peroxide
HNS: Healthy non-smoker
IC: Inspiratory capacity
ICC: Immunocytochemistry
ICS: Inhaled corticosteroid
IL-: Interleukin-
JAK: Janus kinase
KCO: Transfer coefficient
LABC: Lower airway bacterial colonization
LLOQ: Lower limit of quantification
mAb: Monoclonal antibody
mMRC: Modified Medical Research Council dyspnoea scale
MSD®: MesoScale Discovery®
ns: Not significant
PBS: Phosphate buffered saline
PBMC: Peripheral blood mononuclear cell
rh: Recombinant human
RPMI: Roswell park memorial institute
S: Smoker
sIL-6R: Soluble interleukin-6 receptor
SGRQ: St George’s respiratory questionnaire
STAT: Signal transducer and activator of transcription
TCC: Total cell count
TCC/g: Total cell count/gram
TLC: Total lung capacity
6MWT: 6 minute walk test
