# Supplementary material for: Increased levels of soluble interleukin-6 receptor and CCL3 in COPD sputum
Source: Respir Res. 2014 Sep 4;15(1):103. doi: 10.1186/s12931-014-0103-4 (PMC4156958; doi:10.1186/s12931-014-0103-4)
Supplement: Additional file 1: Table S1. — Depicts the demographic details of the 6HNS who donated blood for CD14+ monocyte isolation. [file 12931_2014_103_MOESM1_ESM.docx]

e-Table 1. Demographic details of HNS who donated blood for CD14+ monocyte isolation

| Age (years)* | 30 (25-42) |
| --- | --- |
| Sex (F:M) | 2:4 |
| Pack years smoked | 0 |
| FEV1%^1a^ | 90.8 (4.7) |
| FEV1/FVC%^1^* | 82.6 (71.5-86.6) |
| FEV1%^2a^ | 94.9 (2.5) |
| FEV1/FVC%^2^* | 84.3 (80-92.2) |

This table depicts the demographic details of 6 HNS who donated blood for CD14+ monocyte isolation. ^a^Data is presented as mean (SD). *Data is presented as median (range). ^1^ Pre-bronchodilator. ^2^ Post-bronchodilator.
