# Supplementary material for: Increased levels of soluble interleukin-6 receptor and CCL3 in COPD sputum
Source: Respir Res. 2014 Sep 4;15(1):103. doi: 10.1186/s12931-014-0103-4 (PMC4156958; doi:10.1186/s12931-014-0103-4)
Supplement: Additional file 3: Table S2. — Depicts univariate correlatons between sputum inflammatory cells and sputum cytokines measured in COPD sputum supernatant. [file 12931_2014_103_MOESM3_ESM.doc]

**e**-**Table 2. Univariate correlations of sputum cytokine levels and sputum cell counts in COPD patients**

|  | IL-6 | | sIL-6R | | CCL3 | |
| --- | --- | --- | --- | --- | --- | --- |
|  | r | p | r | p | r | **p** |
| Neutrophil % | 0.2 | 0.15 | 0.1 | 0.42 | -0.02 | 0.88 |
| Macrophage % | -0.2 | 0.18 | -0.2 | 0.14 | -0.04 | 0.76 |
| Eosinophil % | 0.02 | 0.85 | 0.3 | 0.04 | 0.3 | 0.03 |
| Lymphocyte % | -0.3 | 0.04 | -0.1 | 0.50 | 0.03 | 0.83 |
| Sputum TCC/g | 0.1 | 0.38 | 0.6 | <0.0001 | 0.2 | 0.25 |
| Neutrophil TCC/g | 0.2 | 0.21 | 0.5 | <0.0001 | 0.1 | 0.43 |
| Macrophage TCC/g | -0.003 | 0.98 | 0.3 | 0.01 | 0.1 | 0.47 |
| Eosinophil TCC/g | 0.04 | 0.78 | 0.5 | 0.0004 | 0.2 | 0.07 |
| Lymphocyte TCC/g | -0.2 | 0.08 | -0.1 | 0.4 | -0.1 | 0.68 |

(r) represents Spearman Rank correlation coefficient.

Abbreviations used: TCC = Total cell count, TCC/g = Total cell count/gram
