# Supplementary material for: Increased levels of soluble interleukin-6 receptor and CCL3 in COPD sputum
Source: Respir Res. 2014 Sep 4;15(1):103. doi: 10.1186/s12931-014-0103-4 (PMC4156958; doi:10.1186/s12931-014-0103-4)
Supplement: Additional file 4: Table S3. — Depicts comparisons of sputum cytokine levels in COPD patients with chronic bronchitis compared to those without chronic bronchitis. [file 12931_2014_103_MOESM4_ESM.doc]

**e**-**Table 3. Sputum cytokine levels in those COPD subjects with chronic bronchitis compared with non-chronic bronchitis sufferers**

|  | IL-6 (pg/ml) | sIL-6R (pg/ml) | CCL3 (pg/ml) |
| --- | --- | --- | --- |
| Chronic bronchitis  (n = 35) | 90.1  (57.1 - 347.4) | 173.2  (71.5 - 1162) | 152.6  (87.4 - 900) |
| Non-chronic bronchitis  (n = 24) | 82.1  (18.9 - 303.6) | 164.3  (9.2 - 1032) | 99.1  (33.9 - 409.8) |

The table depicts the median (range) of sputum cytokine levels in COPD patients with chronic bronchitis compared with those without chronic bronchitis. Mann-Whitney U tests were performed to establish the statistical significance of any differences between the two groups. P>0.05 for all comparisons.
