# Supplementary figures and images for: Increased levels of soluble interleukin-6 receptor and CCL3 in COPD sputum
Source: Respir Res. 2014 Sep 4;15(1):103. doi: 10.1186/s12931-014-0103-4 (PMC4156958; doi:10.1186/s12931-014-0103-4)

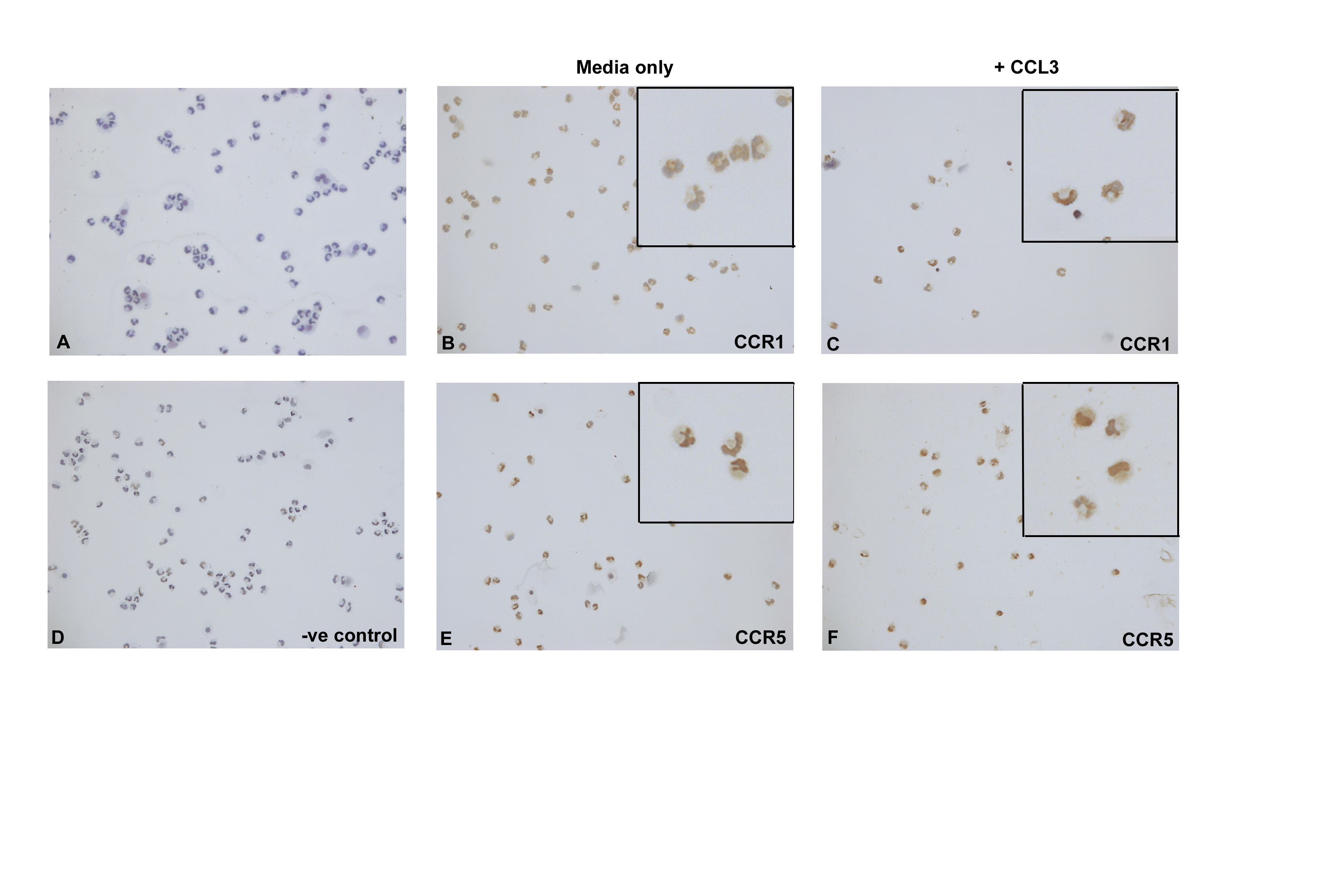

Supplement: Additional file 5: Figure S1. — Depicts immunocytochemistry for CCR1 and CCR5 expression on isolated CD14+ peripheral blood monocytes in the presence/absence of CCL3. [file 12931_2014_103_MOESM5_ESM.jpeg]
